# Supplementary material for: HypE-GA based study on optimal design of standard floor facade windowing of high-rise office buildings facing energy saving in heating, cooling and lighting
Source: PLoS One. 2025 Feb 12;20(2):e0309817. doi: 10.1371/journal.pone.0309817 (PMC11819471; doi:10.1371/journal.pone.0309817)
Supplement: Attachment — Submitted filename: Response to Reviewers.docx [file pone.0309817.s001.docx]

*PLoS One*

Dear editor and reviewers,

Thank you for your valuable comments on this study! I have finished revising the research article, PONE-D-24-03690. Original title: **HypE-GA Based Optimization Design of Facade Window Opening of High-Rise Office Buildings**. Here is my response to the reviewer's comments, a Q&A format.

**To Reviewer #1,**

Q: While the annual cooling, heating, and lighting energy use have been separately observed and analyzed in this case, the overall building performance is also related with and determined by thermal comfort, daylight sufficiency and visual comfort; all of which require a greater multi-objective optimization method. Thus, in that global sense, this paper only offers a more detailed observation on the energy aspect, but not on the whole building performance. Discussion on the impact and relation between energy, thermal, and daylight aspects of the building due to the window design should be provided as well. **At the very least, the title must be adjusted to better reflect the limited scope of performance analyzed in this study.**

A: Indeed, the title should focus on specific performance goals, so the title has been adjusted. the new title is **“HypE-GA based study on optimal design of standard floor facade windowing of high-rise office buildings facing energy saving in heating, cooling and lighting”.** Also, as you said “While the annual cooling, heating, and lighting energy use have been separately observed and analyzed in this case, the overall building performance is also related with and determined by thermal comfort, daylight sufficiency and visual comfort; all of which require a greater multi-objective optimization method.” In our past research, we have also tried to consider coupling more building performance objectives, such as natural daylighting, indoor thermal comfort, and have published our research results in international conferences. Find it at <https://doi.org/10.1117/12.2646586>

Q: **In Eq. (1), what do an1, an2, an3 represent?**

A: In order to express the meaning of the formula more clearly, we have made some adjustments to the formula, especially in the letters. an1, an2, an3 are changed to,, respectively.,,are weighting factors of,respectively. New Eq. (1):

(1)

Q: **Uncommon abbreviations such as AC, AH, AL must be defined first in the text, despite having listed in the List of Abbreviations in p. 1.**

A: All phrases in abbreviated form, whether in the abstract section or in the main text, are given the full name on their first appearance.

Q: In the explanation of Eqs. (1) and (2), the symbol (kW h m^-2 a^-1 ) is just the unit of the performance indicator, which is the Energy Use Intensity (EUI). **Thus, the term "min (EUI)" should be employed instead.**

A: New Eq (1) and Eq (2), respectively, the symbol (kW h m^-2 a^-1) has been changed.

Where in Eq (1),

$f_{1}(X)=AC\left(\begin{matrix} WWR & WH & SH \end{matrix}\right)\to\min\left(EUI\right)$,‬‬‬‬

$f_{2}(X)=AH\left(\begin{matrix} WWR & WH & SH \end{matrix}\right)\to\min\left(EUI\right)$,‬‬‬‬

$f_{3}(X)=AL\left(\begin{matrix} WWR & WH & SH \end{matrix}\right)\to\min\left(EUI\right)$.

Where in Eq (2),

$$F_{2}(X)=f_{1}(X)+f_{2}(X)+f_{3}(X)=\left\{AC\left(\begin{matrix} WWR & WH & SH \end{matrix}\right)+AH\left(\begin{matrix} WWR & WH & SH \end{matrix}\right)+AL\left(\begin{matrix} WWR & WH & SH \end{matrix}\right)\right\}\to\min\left(EUI\right)$$

Q: **In Fig. 3, the length and width of the floorplan should be given in the illustration.**

A: See new Fig 4 followed.

**Fig 4. standard floor office unit model**

Q: **Why are the weather data (TMY) still taken from the year 2005? Any other reasons? How different are the data, compared to more recent weather data?**

A: Regarding the reasons for choosing the meteorological data for 2005, we have made further additions to the original basis. “The typical meteorological year (2005) data of Yantai Longkou weather station were read by LB. This is a Chinese Standard Weather Data (CSWD) researched and developed by the Meteorological Information Center of the China Meteorological Administration in conjunction with the Department of Building Science of Tsinghua University, which was updated in 2005 and is still commonly used in studies of building simulation. **In addition, in 2005, the Yantai region was hit by a huge snowstorm, and the lowest temperature broke the record within 50 years, which was called the year of extremely low temperature, so the meteorological data of that year is particularly important for studying energy conservation in heating demand buildings in northern China**.” In addition, it should also be noted that the meteorological data for recent years have been relatively stable compared to 2005, and are therefore not as representative as they could be. For heating buildings in the northern cold zone, it is necessary to consider the historical extreme cold years as the meteorological conditions for simulation.

Q: **The Hype-GA assignment settings (Tables 5 and 7) must be written in the Method section, not in the Results. What is the reference for using that settings?**

A: Tables 5 and 7 have been replaced by new Tables 1 and 2, which located **in the Optimization algorithm subsection of the Methodology section.** The setting of the algorithm parameters in the table is also explained. “The operating parameters, including Elitism, Mutation Rate, Mut.Probability, Crossover Rate, Population Size and Max Generations, are very critical because different settings of parameters will have a large impact on the efficiency of HypE-GA. In the optimal process, Elitism gives at least 50% new solutions that are bred out of the Elite instead of the entire pool. The lower Crossover Rate, the less information is exchanged between individuals, resulting in undercharacterised new solutions, so a value of 0.4 ~ 0.99 is recommended. A low Mutation Rate means little changes to the parameters’ values, multiplied by Mut.Probability is generally recommended to take a value of 0.0001 ~ 0.1. Population Size and Max Generations directly affects the efficiency of HypE-GA, which can be set a value according to the case complexity in 10 ~ 50 and in 10 ~ 200, respectively. In this case study, based on the above principles, operating parameters of single-objective HypE-GA are shown in Table 1, and that of multi-objective HypE-GA are shown in Table 2.”

**Table 1. Single-objective HypE-GA** **assignment**

| **Sub-items** | Elitism | Mutation Rate | Mut.Probability | Crossover Rate | Population Size | Max Generations |
| --- | --- | --- | --- | --- | --- | --- |
| **Settings** | 0.5 | 0.01 | 0.2 | 0.6 | 30 | 20 |

**Table 2. Multi-objective HypE-GA assignment**

| **Sub-items** | Elitism | Mutation Rate | Mut.Probability | Crossover Rate | Population Size | Max Generations |
| --- | --- | --- | --- | --- | --- | --- |
| **Settings** | 0.5 | 0.01 | 0.2 | 0.5 | 50 | 100 |

Q: **Prior to optimization, there is typically sensitivity analysis conducted to identify the most influential input variables on each output variable (performance indicator). Please consider doing this analysis.**

A: In our previous study, we have done the sensitivity analysis of WWR,WH,SH with AH,AC,AL and AT as the building performance targets, and get some conclusions like different factors have varied influences on AT under different orientations: WWR_S_ > WWR_N_ > WWR_E_ > WWR_W_; WH_E_ > WH_S_ > WH_N_ > WH_W_; SH_S_ > SH_E_ > SH_N_ > SH_W_. Finding it at https://link.cnki.net/doi/10.13951/j.cnki.37-1213/n.210112.

Q: **Please provide units for AC, AH, AL in Tables 6 and 8. The term "Pareto" in those tables is misleading, because in a pair of two objectives, there can be many Pareto optimal solutions, instead of only one. What does "Pareto" there actually mean?**

A: Units are displayed in the notes below the Tables 6, which has been changed as new Tables 7. In order to better express the correspondence between the data in Table 8 and the scatterplot, we have merged them into what is new Figure 6, and the units are labeled in the figure .

**Fig 6. Building performance simulation results of office1 ~ office8 under 100 iterations**

Regarding the interpretation of pareto, we apologize for not explaining it in our previous article, which led to a misunderstanding on the part of our readers. Now, we make the explanation in single-objective optimization simulation and multi-objective optimization simulation respectively, so that the reader understands the meaning of pareto in single/objective optimization results. We have made additions in Results and discussion section, “**During the single-objective building performance optimization process, the convergence result of the operation tends to be unique and there exists a unique Pareto optimal solution**. Table 7 shows the window opening states when the Pareto solution is reached for different building performance objectives.” and “building energy efficiency research involves synergistic effects between multiple performance objectives, and thus the coupling effects of AC, AH, and AL in each office unit need to be further investigated through a multi-objective optimization approach. **In this process, it is generally difficult to find the solution that is optimal for all building performance objectives, i.e., there exists a Pareto set solutions.** The location of the optimal Pareto solution for each building performance objective in the end-generation Pareto solution set is found by color gradient clustering analysis and marked with A, B, C and D. Fig. 5 shows the window opening status of office1 ~ office8 facades under multi-objective coupled optimization under 100 iterations. **The four labels, A, B, C and D, are used to denote the Pareto optimal solution of AH, AC, AL, and AT for each office unit, respectively.**”

Q: **What is the computation time for each simulation using HypE-GA in study? How is it compared to simulation with other algorithms?**

A: Regarding computation time for each simulation using HypE-GA in study, I'm sorry we haven't kept statistical records. From memory we calculate it in about 2 minutes a time, but it takes longer to iterate it once. In single-objective optimization, 20 iterations total about 260 hours; in multi-objective optimization, 100 iterations total about 730 hours.The fact that we treat each office unit as a separate run file, and therefore cannot run in parallel, adds significantly to our time costs. Single- and multi-objective run files we will submit with the article to ensure the operationalization and generalization of our design process.

HypE-GA has unique advantages over the three evolutionary algorithms with which we are most familiar. We add in the paper “HypE-GA uses Monte Carlo simulations to approximate the exact hypervolume values. The main idea is that the actual metric value is not important, but the ranking of solutions induced by the hypervolume metric is important. Thus, hypervolume-based search not only solves multi-objective problems, but also flexibly adjusts the running time. Bader and Zitzler showed HypE-GA was used for more accuracy and speed than similar algorithms, including Strength Pareto Evolutionary Algorithm Ⅱ (SPEA-Ⅱ) , Nondominated Sorting Genetic Algorithm Ⅱ (NSGA-Ⅱ) and Indicator-Based Evolutionary Algorithm (IBEA), in the optimization process in cases with more than three fitness functions. The HypE-GA was matched with HypE Reduction convergence mechanism and HypE Mutation mechanism. Hype Reduction stipulated how to truncate a Pareto front solutions to fit the result size when it is too big. HypE Mutation applied mutation and recombination operators to generate N offspring to simulate the diversity of iterative processes. Therefore, HypE-GA is an appropriate way to solve single-objective or multi-objective optimization problems in this study.” Find it at https://www.sci-hub.yt/10.1162/evco_a_00009

Q: **Discussions are needed in terms of comparison with many relevant studies on the topic of window design optimization, though not necessarily those using GA. What is then the recommended direction for follow-up studies?**

To discuss future work, the findings provide the following recommendations and implications.

(1) Based on the same methodology, the energy-saving optimization strategies for façade casement windows in different climatic zones and building types in China can be further investigated, so that on the one hand the optimization technology can be promoted, and on the other hand, the performance of local buildings can be improved.

(2) For the single façade window opening form of a building, the building's thermal and optical performance (including natural lighting, thermal comfort, window opening behavior, natural ventilation and airtightness, etc.) needs to be further investigated. In addition to this, the optimization of building performance under diverse window opening forms can be further investigated.

(3) Under certain conditions, optimization results in the best design strategy. It is still necessary to further consider more objective conditions (thermal properties and construction methods of the facades and windows themselves) and subjective awareness (professional concepts of the designers and needs of the users). In addition, the research system is grounded in the stages of early design and late modification, based on building models-ruled and basic simulation conditions, and its feasibility is yet to be verified in real cases.

**To Reviewer #2,**

Q: **The paper lacks detailed descriptions of the underlying scientific principles and theoretical frameworks guiding the optimization algorithms used (HypE-GA). Without a clear theoretical foundation, it is challenging for readers to assess the validity and appropriateness of the optimization methods employed.**

A: HypE-GA is one of the GA's that are characterized by advantages in computational processes with many nonlinearities formed by high-dimensional design parameters and three and more optimization objectives. Firstly, in the process of many iterative calculations, there will not be a bureau early convergence Pareto solution set, but the generation is more excellent to develop. Secondly, the use of Monte Carlo simulation to approximate the exact hypervolume values improves the efficiency and accuracy of model data optimization. Third, the ranking of monotonicity excellent solutions is always maintained in the results of each generation of optimization, which also saves the time and effort of manual data processing.

Q: **The technical details of the HypE-GA algorithm, such as convergence mechanisms and mutation strategies, are provided, but there is insufficient explanation or justification for why these specific settings were chosen. Without a clear rationale, readers cannot evaluate the technical accuracy of the optimization process.**

A: The paper adds the connotation of HypE Reduction convergence mechanism and HypE Mutation mechanism. i.e., “The HypE-GA was matched with HypE Reduction convergence mechanism and HypE Mutation mechanism. The set of objective value vectors corresponding to each solution in the Pareto Set is called the Pareto Front. Hype Reduction stipulated how to truncate a Pareto front solutions to fit the result size when it is too big. HypE Mutation applied mutation and recombination operators to generate N offspring to simulate the diversity of iterative processes.” And “A low Mutation Rate means little changes to the parameters’ values, multiplied by Mut.Probability is generally recommended to take a value of 0.0001 ~ 0.1.” Because of the possibility of mutations in the N new individuals produced by crossover, this is more in line with the laws of nature, but in order to ensure a smooth iteration, generally go to a very small value, 0.0001~0.1.

Q: **The paper lacks context regarding the novelty or significance of the findings. While the optimization results are presented, there is no discussion of how they advance the current understanding of facade windowing design in high-rise office buildings. Without this context, it is difficult to assess the representativeness of the results for the broader scientific community.**

A: **At the end of this part of the introduction**, we add a description of the innovative and contextual nature of this study, explaining that this type of research is topical at the moment and that the innovations focus on the design methodology. “Nowadays, the green concept in green building, urban renewal and remodeling has attracted much attention.In previous architectural research and practice, there are numerous software for simulating building energy efficiency, but their single functionality and weak interaction between software cannot fully meet the design needs of architects. Therefore, on the one hand, to solve the problems of unfriendly multi-platform interactivity and low optimization efficiency, especially for design projects based on multi-objective optimization. On the other hand, to provide architects with objective and fast decision support for early design and later remodeling of façade windowing. Studying the relationship between windowing design and single/multi-objective building performances in high-rise office buildings in cold region applying through LB + HB and Hypervolume Estimation Genetic Algorithm (HypE-GA), which becomes an innovative aspect that differs from above studies.”

The novelty and accuracy of the results are expected to be further verified through the practice of engineering projects. Not only will the results of this study be verified, but also more design parameters and more performance objectives will be incorporated to optimise the indoor environment of the building, as we mentioned **in this section of our future work**. “(2) For the single façade window opening form of a building, the building's thermal and optical performance (including natural lighting, thermal comfort, window opening behavior, natural ventilation and airtightness, etc.) needs to be further investigated. In addition to this, the optimization of building performance under diverse window opening forms can be further investigated. (3) Under certain conditions, optimization results in the best design strategy. It is still necessary to further consider more objective conditions (thermal properties and construction methods of the facades and windows themselves) and subjective awareness (professional concepts of the designers and needs of the users). In addition, the research system is grounded in the stages of early design and late modification, based on building models-ruled and basic simulation conditions, and its feasibility is yet to be verified in real cases.”

Q: **Although the optimization settings and results are provided, there is insufficient detail on the simulation methods and input parameters used.**

A: The article provides the data for each environment setting input, but we provide the details of the operation in the programme documentation. The following details of the main sections are available for inspection.

**Fig a.** **Energy consumption model**

**Fig b.** **Façade windowing**

**Fig c. External wall construction and its material properties**

**Fig d. Internal wall construction and its material properties**

**Fig e. Floor, roof and ceiling construction and its material properties**

**Fig f. Floor, roof and ceiling construction and its material properties**

**Fig g. Operation schedules of occupancy rate**

**Fig h. Operation schedules of heating**

**Fig g. Operation schedules of cooling**

**Fig g. Operation schedules of lighting**

**Fig g. Operation schedules of equipment**

**Fig h. Operation schedules of infiltration**

**Fig i. Daylight setting**

**Fig j. Other loads setting**

**Fig k. HVAC setting**

Q: **The phrase "This paper investigates..." in the conclusion section represents self-definition. Such phrases should be avoided as they may imply a lack of objectivity and scientific detachment.**

A: The formulation of this place has been adjusted. “Taking the LB + HB platform and HypE-GA to study on intrinsic relationship between building performances and façade windowing design of standard floor of high-rise office buildings in cold region , and providing support for architects engaged in this aspect. The following summarizes several conclusions of this research.”

Q: **The conclusions drawn in the paper lack sufficient empirical support from the data presented. There is a need for more explicit connections between the optimization results and the conclusions drawn regarding the impact of facade windowing on building energy performance.**

A: The results of the single-objective optimisation and the multi-objective optimisation were obtained by analysing the data data in the new Table 7 and Figure 5, respectively. Both single-objective optimisation and multi-objective optimisation have concluded that WWR is the dominant factor affecting AT, so we have given the proposed WWR values for each façade orientation in the optimisation results by summing over averaging. The next conclusion is that larger values of WH and SH are better, which is a directional conclusion. If our optimal solutions in Table 7 and Fig. 5 are not used, then WH and SH take the limiting values for a given WWR.

Q: **The paper utilizes artificial intelligence (AI) techniques, specifically the HypE-GA algorithm, for optimization purposes. However, the paper does not clearly specify how AI is integrated into the research methodology, limiting the transparency and reproducibility of the approach.**

A：In the new Fig 3, we shed light on the AI technology, HypE-GA's operator Octopus. i.e., “A plug-in Octopus (V4.0) with HypE-GA, which connects design variables at the input (G port) and building performance metrics as evaluation targets at the output (O port), drives the energy model with iterative operations (see Fig 3). Double-click G to expand the Octopus operator, which is divided into four regions, R1~R4. R2 is the main view region, and the R1 region has two functions, one is to regulate the size and the state of the main view and the solution, and the other is to observe the state of each iteration, such as calculating once time, the number of dominated and undominated, and the size of the evolutionary pool. R3 region is to observe the convergence degree, parameter diversity and coordinate naming of performance goals. R4 region is to set the parameters of HyeE-GA and select the convergence mechanism, and mutation mechanism.”

**Fig 3. Octopus operator interface composition**

Above, are all my responses to the two reviewers, thank you again for your valuable comments.

Best regards,

Weixiang Zhang
