# Supplementary material for: HypE-GA based study on optimal design of standard floor facade windowing of high-rise office buildings facing energy saving in heating, cooling and lighting
Source: PLoS One. 2025 Feb 12;20(2):e0309817. doi: 10.1371/journal.pone.0309817 (PMC11819471; doi:10.1371/journal.pone.0309817)
Supplement: Attachment — Submitted filename: Response to Reviewers.docx [file pone.0309817.s002.docx]

*PLoS One*

Dear editor and reviewers,

Thank you for your valuable comments on this study! I have finished revising the research article, PONE-D-24-03690. Paper title: **HypE-GA based study on optimal design of standard floor facade windowing of high-rise office buildings facing energy saving in heating, cooling and lighting**. Please pay special attention to the following additions to my grant fund: The authors disclosed receipt of the following financial support for the research, authorship and publication of this article: The research described in this paper was funded by Yantai Institute of Technology Youth Foundation Program (2023QNJJ03), Yantai Institute of Technology Academic Team Program (2022XSTD04) and Shandong Social Science Planning Research Program (22CWYJ35). The funders had no role in study design, data collection and analysis, decision to publish, or preparation of the manuscript.

In addition, here is my response to the reviewer's comments, a Q&A format. Sections in red indicate additions or modifications as the below.

**To Reviewer #1,**

Q: **The opening sentence in the Abstract ("Taking the LB + HB platform...") is a bit difficult to understand and is not commonly found as a typical abstract opening sentence.** **Perhaps the wordings should be rephrased. A general background shall be provided, followed with the knowledge gap and aims or objectives of the study.**

A: The quantitative design on area and location of building façade’s windows has a significant impact on interior light and heat environment, which is also very instructive for preliminary and remodeling design of buildings. However, previous studies paid more attention to the thermal insulation construction and shading based on design parameters from the perspective of designers, but neglected the fact that the geometric properties of the windows themselves are equally important for building energy efficiency. Secondly, the weak interactivity and algorithmic limitations of traditional simulation platforms prevent rapid access to ideal design strategies. Therefore, this paper takes the standard floor of a high-rise office building as the research object in cold region－Yantai, facing façade windowing design, the three building performance objectives of each office unit－Annual Cooling Energy Consumption (AC), Annual Heating Energy Consumption (AH) and Annual Lighting Energy Consumption (AL)－are simulated and single/multi-objective optimized by relying on Ladybug and Honeybee (LB + HB) platform and Hypervolume Estimation Genetic Algorithm (HypE-GA) to obtain the genome of Pareto－Window-to-Wall Ratio (WWR), Window Height (WH) and Sill Height (SH)－at the lowest of each performance objective in order to determine the most energy-efficient façade windowing expression. The results show that AH and AC, their sum of quantities remains stable, are main energy consumption sources of office buildings, while the change of AL is more likely to have an impact than the others’ on Annual Totaling Energy Consumption (AT). The analysis points out that different windowing strategies can be adopted for different performance objectives. To reduce AC, priority is given to windowing on the east and north facade, with East Window-to-Wall Ratio (WWRE) at 0.2 ~ 0.3 and North Window-to-Wall Ratio (WWRN) at 0.3 ~ 0.5; to reduce AH, windows on the west and north facade should not be opened, and the remaining facades should be opened in small areas; to reduce AL, WWR> 0.7 is appropriate for each facade, and should be considered to matching a higher SH or WH; From AT, the average WWR in the single-objective and multi-objective optimization results are similar, so it is suggested that the WWR of each facade of office buildings in Yantai area is WWRE=0.47, North South Window-to-Wall Ratio (WWRS)=0.46, West Window-to-Wall Ratio (WWRW)=0.18 and WWRN=0.54. In addition, this paper proposes a method that can quickly find the Pareto optimal solution by clustering analysis on optimized results through Origin in multi-objective HypE-GA optimization study.

Q: **in some of their responses, the authors refer to their previous works, but those works are not mentioned or properly cited in the text. We should not assume that the readers would have been familiar with those previous works, so the current manuscript should concisely describe and cite them.**

A: **In the authors' previous studies,** two parts of the work were specifically done. Not only verified that window geometric parameters have the greatest sensitivity to building performance, but also remedied the above two research shortcomings. In the first part, the multicollinearity problem between high-dimensional design parameters and multi-objective building performance (building energy consumption, daylighting, indoor thermal comfort) was solved by principal component analysis. It was found that only 12 of the preset 15 parameters passed the KMO Sampling Suitability Quantity and BARTLETT sphericity test, and they were classified into four principal components: Building Body (Bay, Depth and WWR), Windowing Geometry (Window Height, Sill Height and Window Horizontal Distance), Window Properties (K-value, Solar Heat Gain Coefficient and Visual Transmittance), and Facade Properties (Wall Height, Plane Corner, External Wall R-Value), and the ensuing weight calculations yielded that the weights of each principal component were 0.41, 0.268, 0.188, and 0.134 in that order, thus proposing the principal component linear function assessment model. In the design of buildings with Bay and Depth, the Windowing Geometry with WWR is the most sensitive type of design parameters for building performance **[24]**. In the second part, the sensitivity of window geometric parameters to building energy consumption (AC, AH, AL and AT) was investigated by variance calculation. It is shown that WWR is the dominant factor affecting indoor AC and AH in the design of facade windowing, and larger values of WWR, WH and SH within a certain range are preferable in terms of AT reduction. The best orientation for window-opening is south, east, north, and west in a sequence when WWR is in the range of 0.3 to 0.7. Different factors have varied influences on AT under different orientations: WWR_S_ > WWR_N_ > WWR_E_ > WWR_W_；WH_E_ > WH_S_ > WH_N_ > WH_W_；SH_S_ > SH_E_ > SH_N_ > SH_W_**[25]**。

These are the research methodology and some of the conclusions from previous studies, in this paper in references [24], [25]. In addition, there are images of the simulation program mentioned in the last reply letter that have been shared as modelling details in a zip package, all uploaded to the system.

Q: **Definitions of Window Width (WW) and Window Edge Distance (WED) should be provided with illustrations.**

A: Additional information in the paper “Horizontally, the WW is the width of the window opening and the WED is the distance from the side of the window opening to the demarcation line of the office unit.”

**Fig 4. Standard floor office unit model**

Q: In the multi-objective optimization, in a given pair of two conflicting objectives, there can be many Pareto optimal solutions (in plural, with 's'). These optimal solutions altogether are called the Pareto optimal set, or just Pareto set, or Pareto front (in singular). Thus, be careful and consistent when referring to those terminologies. For instance, in line 306: "there exists a Pareto set solutions" is not appropriate. It should be "there exists a Pareto optimal set", or "there exists a set of Pareto optimal solutions" (although the former is preferrable).**In lines 311 and 314: "Pareto front solutions" should be "the Pareto set". Please check for any similar occurrences.**

A: Forms of words have been modified in the paper. “Optimal results were presented in Table7 on façade windows of each office unit under single-objective optimization. In fact, building energy efficiency research involves synergistic effects between multiple performance objectives, and thus the coupling effects of AC, AH, and AL in each office unit need to be further investigated through a multi-objective optimization approach. In this process, it is generally difficult to find the solution that is optimal for all building performance objectives, i.e., there exists a set of Pareto optimal solutions. The location of the optimal Pareto solution for each building performance objective in the end-generation Pareto set is found by color gradient clustering analysis and marked with A, B, C and D. Fig. 6 shows the window opening status of office1 ~ office8 facades under multi-objective coupled optimization under 100 iterations.

It is not easy to obtain optimal solution from Pareto front solutions in the plugin Octopus, so importing genomes and data of all solutions into Origin, redraw 3D scatter plot, and establish chromatographic simple clustering according to the numerical magnitude of AT, which can quickly find the optimal solution from the Pareto set, and analyze the optimal state of individual performance targets. In addition, genomes and target groups corresponding to target points are fed back to the simulation system for testing to confirm the reliability of a design solution.”

Q: **A dedicated Discussion or General Discussion section is recommended, prior to the Conclusion section. See comment from the previous review regarding comparison with many relevant studies on this topic, and recommended direction for follow-up studies.**

**A:** A **Discussion subsection** was added before the **Conclusion section**, where the progress made in the research results over the previous research articles was discussed, as well as the problems that still exist. Even future research directions are discussed. **“**From the above results of single-objective optimization and multi-objective optimization of building energy consumption, the WWRs-valued of four different windowing orientations are showed very close. In general, designers need to consider buildings’ AT more energy efficient, so necessarily refers to the design method of multi-objective optimization. In previous studies, to design more energy efficient windowing solutions for buildings, it is necessary to consider the climate, building types, and constructions and materials of three components, including wall, window frame and glass, but the equally important window geometry factors (WWR, WH, SH, etc.) are seldom considered, or only WWR is highlighted **[14].** This study will limit a portion of the building's geometric factors (window directions, bay and depth) and focus on the role of geometric factors (WWR, WH, SH) related to windowing location on the façade on the building's energy efficiency, and it also further improves studies on the windowing geometric factors **[16]**. This design process is of strong practical significance, both in the early design of façade windowing and in the later reconstruction of façade windowing. Yantai was chosen as the target city for this study, which represents only a very small portion of the cold region, but this research methodology is regionally unrestricted. For designers, the Grasshopper simulation platform in Rhino is more user-friendly, especially surpassing traditional simulation platforms (EnergyPlus, DesignBuilder, DeST, etc.) in terms of human-computer interactivity and diversity of visual representations. In addition, HypE-GA provides more excellent solutions in the process of coupled simulation and optimization of multiple building performances, which share the effort required to be invested in the previous designers' extrapolation and decision-making process compared with the traditional intelligent optimization algorithms **[20,44]**. Although HypE-GA improves the computation rate, it still takes a long time. It still consumes a lot of computational time and statistical effort if faced with more complex projects, i.e., projects involving more design parameters and coupling more building properties. To discuss future research efforts, the findings suggest the following directions:

(1) Based on the same methodology, the energy-saving optimization strategies for façade casement windows in different climatic zones and building types in China can be further investigated, so that on the one hand the optimization technology can be promoted, and on the other hand, the performance of local buildings can be improved.

(2) For the single façade window opening form of a building, the building's thermal and optical performance (including natural lighting, thermal comfort, window opening behavior, natural ventilation and airtightness, etc.) needs to be further investigated. In addition to this, the optimization of building performance under diverse window opening forms can be further investigated.

(3) Under certain conditions, optimization results in the best design strategy. It is still necessary to further consider more objective conditions (thermal properties and construction methods of the facades and windows themselves) and subjective awareness (professional concepts of the designers and needs of the users). In addition, the research system is grounded in the stages of early design and late modification, based on building models-ruled and basic simulation conditions, and its feasibility is yet to be verified in real cases.”

**To Reviewer #2,**

Q: **The study focuses predominantly on energy consumption (AC, AH, AL) without adequately integrating other crucial aspects of building performance such as thermal comfort, daylight sufficiency, and visual comfort. This limitation reduces the holistic applicability of the findings in real-world scenarios where occupant comfort is equally important.** **the absence of statistical validation and sensitivity analysis weakens the robustness of the findings from a scientific standpoint.**

A: The focus of this article's research is to observe the effect of window geometric features on AC, AL, and AH through their design parameters and to obtain an energy efficient window opening strategy. In the authors' previous studies, the intrinsic link between window opening (considering the physical properties of the adjacent envelope) on lighting, energy consumption, and thermal comfort in building interiors has been investigated on the one hand, as detailed in Ref. [24].On the other hand, the sensitivity of the design parameters(WWR, AH, SH) to AC, AL, and AH has been studied, and the results of their research are mentioned in the introductory section of this article, as detailed in Ref. [25]. In the **Introduction Section** of this article, a review of the important conclusions of the authors' previous studies in these two aspects.

Q: **The study uses Typical Meteorological Year (TMY) data from 2005 without justifying why this dataset was chosen over more recent data. Climate data can significantly affect energy performance simulations, and using outdated data might not reflect current or future conditions accurately.**

A: Regarding the reasons for choosing the meteorological data for 2005, we have made further additions to the original basis. “The typical meteorological year (2005) data of Yantai Longkou weather station were read by LB. This is a Chinese Standard Weather Data (CSWD) researched and developed by the Meteorological Information Center of the China Meteorological Administration in conjunction with the Department of Building Science of Tsinghua University, which was updated in 2005 and is still commonly used in studies of building simulation. In addition, in 2005, the Yantai region was hit by a huge snowstorm, and **the lowest temperature broke the record within 50 years, which was called the year of extremely low temperature**, so the meteorological data of that year is particularly important for studying energy conservation in heating demand buildings in northern China.” In addition, **it should also be noted that the meteorological data for recent years (2006 to 2022) have been relatively stable compared to 2005, and are therefore not as representative as they could be**. For heating buildings in the current northern cold zone, it is necessary to consider the historical extreme cold years as the meteorological conditions for simulation.

Q: **Some methodological details are vague, such as the explanation of key parameters in equations and the rationale behind specific optimization choices.**

A: The key parameters and optimization principles in the formulation have been further supplemented in the paper. The calculation models for single-objective and multi-objective optimization of this paper, Eq (1) and Eq (2), respectively, are as follows:

(1)

$F_{2}(X)=min\left(\begin{matrix} f_{1}(X) & f_{2}(X) & f_{3}(X) \end{matrix}\right)=\left[\begin{matrix} a_{1} & b_{1} & c_{1} & C_{1} \\ a_{2} & b_{2} & c_{2} & C_{2} \\ a_{3} & b_{3} & c_{3} & C_{3} \end{matrix}\right]\left[\begin{matrix} x_{1} & x_{2} & x_{3} \end{matrix}\right]^{\top}$ (2)

Where,takes 1, 2 or 3，,,,are weighting factors of, respectively,is a constant. The range of values for each design parameter (WWR, WH and SH) is weighted. In Eq (1), the single-objective optimization process is infinitely close to a unique optimal solution, and in Eq (2), multiple optimal solutions will occur in multi-objective coupled optimization.

Where in Eq (1), AC, AH and AL are executed for single objective optimization respectively. As below:

$f_{1}(X)=AC\left(\begin{matrix} WWR & WH & SH \end{matrix}\right)\to\min\left(EUI\right)$,‬‬‬‬

$f_{2}(X)=AH\left(\begin{matrix} WWR & WH & SH \end{matrix}\right)\to\min\left(EUI\right)$,‬‬‬‬

$f_{3}(X)=AL\left(\begin{matrix} WWR & WH & SH \end{matrix}\right)\to\min\left(EUI\right)$.

Where in Eq (2), AC,AH and AL are executed simultaneously for multi-objective optimization. As below:

$$F_{2}(X)=f_{1}(X)+f_{2}(X)+f_{3}(X)=\left\{AC\left(\begin{matrix} WWR & WH & SH \end{matrix}\right)+AH\left(\begin{matrix} WWR & WH & SH \end{matrix}\right)+AL\left(\begin{matrix} WWR & WH & SH \end{matrix}\right)\right\}\to\min\left(EUI\right)$$

Q: **Uncommon abbreviations such as AC, AH, and AL should be defined clearly when first mentioned in the text.**

A: In the paragraph in which it first appears in the text, it is added “The energy consumption quantities counted in the simulation study in this paper come from the equipment terminal and do not consider other losses. The AH and AC are from the HVAC system terminals and the AL is from the lighting system terminals.”

Q: **Figures such as floor plans should include dimensions for better understanding. Additionally, the clarity and labeling of charts and graphs could be improved to enhance interpretability.**

A: Related dimensions, dedicatedly see Fig 4.

**Fig 4. Standard floor office unit model**

There is a capacity limit for uploading files, which may result in images being rendered slightly lower. The original high-resolution images are uploaded in their entirety as a zip archive for review.

Above, are all my responses to the two reviewers, thank you again for your valuable comments.

Best regards,

Weixiang Zhang
